# Anaplastic thyroid carcinoma combined with sclerosing mucoepidermoid carcinoma with eosinophilia: A case report

**DOI:** 10.1097/MD.0000000000022783

**Published:** 2020-10-16

**Authors:** Ruiqi Mao, Lifang Shi, Wei Yan, Wenli Li, Baicheng Li, Xinjun Li

**Affiliations:** Department of Pathology, Binzhou People's Hospital of Shandong Province, Binzhou, China.

**Keywords:** anaplastic thyroid carcinoma, BRAF V600E, differential diagnosis, sclerosing mucoepidermoid carcinoma with eosinophilia, thyroid

## Abstract

**Rationale:**

Anaplastic thyroid carcinoma (ATC) is a rare highly aggressive thyroid malignancy. Thyroid sclerosing mucoepidermoid carcinoma with eosinophilia is also a rare low grade malignant thyroid neoplasm. To date, comorbidity of these 2 tumors in the thyroid gland has not been reported in the English literature.

**Patient concerns:**

Here, we present a case of a 67-year-old women with a 6-month history of mass of left neck. She complained of a painless mass in the right neck.

**Diagnoses:**

Based on histopathological examination of H&E stained sections, immunohistochemical staining assay and molecular tests, the patient was diagnosed with ATC combined with sclerosing mucoepidermoid carcinoma with eosinophilia.

**Interventions:**

The patient underwent radical surgery for thyroid cancer.

**Outcomes:**

No complications, local recurrence or metastases were observed during a 1 year and 3 months follow-up after surgery.

**Lessons:**

To the best of our knowledge, this is the first case report on ATC combined with sclerosing mucoepidermoid carcinoma with eosinophilia in the English literature. This condition can be easily misdiagnosed during thyroid fine needle cytology. Clinicians should perform morphological examination, immunohistochemistry and molecular tests on resected specimen to make a definitive diagnosis.

## 1 Introduction

Anaplastic thyroid carcinoma (ATC) is a rare thyroid malignancy characterized by undifferentiated follicular thyroid cells. This condition accounts for 0.5% to 5% of all thyroid carcinomas.^[1,2]^ ATC is prevalent in females and elderly subjects. Prevailing evidence suggests that ATC is likely to evolve from differentiated thyroid cancer.^[3]^ ATC is the most aggressive type of thyroid cancer, with a mortality rate exceeding 90% after diagnosis.^[2,4]^ Due to the aggressive nature of the disease, accurate and prompt diagnosis is critical to determine appropriate treatment options. Thyroid sclerosing mucoepidermoid carcinoma with eosinophilia was first described by Chan et al in 1991.^[5]^ It is a rare and still enigmatic thyroid neoplasm in adults that presents on average in the fifth decade with a female predilection. It often occurs in the histological background of Hashimoto thyroiditis. Here we discuss a 67-years old women who presented with a mass in the left lobe of the thyroid. She was diagnosed by morphological, immunohistochemistry and molecular tests with thyroid ATC combined with sclerosing mucoepidermoid carcinoma with eosinophilia. This is the first case report of this kind.

## 2 Case presentation

The patient presented with a 6-month history of a painless mass of the left neck. On physical examination, bilateral neck was asymmetry and a firm, fixed mass which could be touched on the left neck. There was no enlargement of the cervical lymph nodes. Ultrasonography revealed a 3.8 cm well-defined hypo-echoic nodule at the left thyroid region with branching blood flow signal. Cervical enhanced computed tomography (CT) confirmed tumors in the left lobe and isthmus of thyroid, and thus malignant lesions were suspected. No other nodules were found in further Emission CT examination.

**Figure 1:**
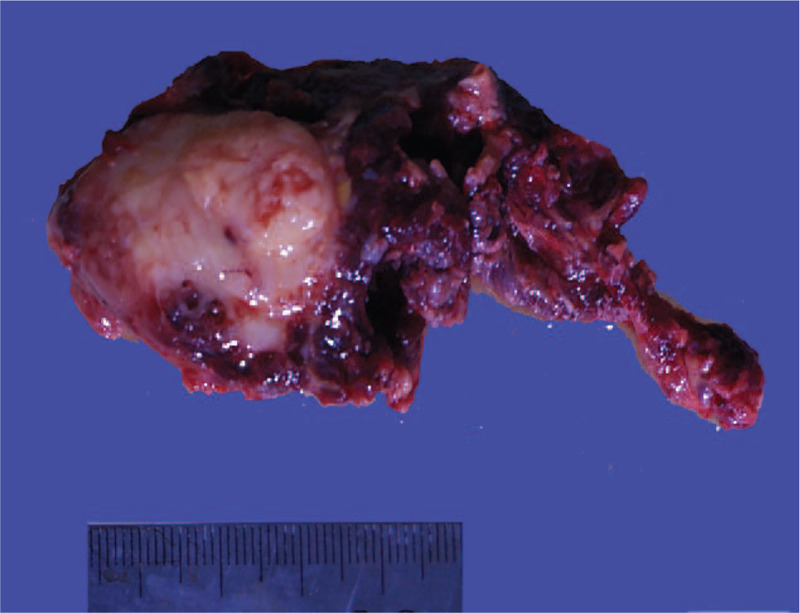
The tumor showing solid mass with gray-white and gray-red in color and 3.5 cm in diameter.

**Figure 2:**
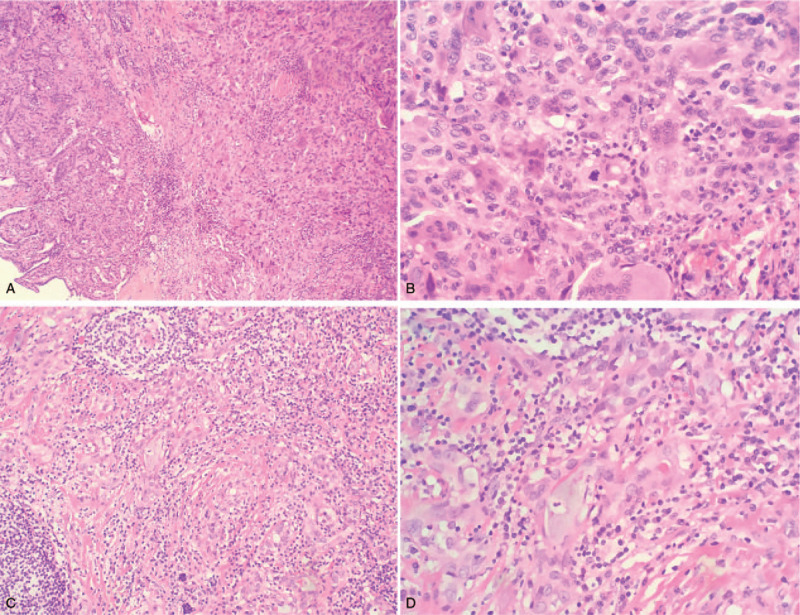
Morphologic features of thyroid mass on hematoxylin-eosin stain. (A) The tumor shows solid nodule with scatter osteoclast giant cell-like multinucleated cells and papillary thyroid carcinoma is on the left (original magnification × 100). (B) The tumor is composed of monocyte-like cells with mitosis and scatter osteoclast giant cell-like cells (original magnification × 400). (C) Another area of the tumor shows lumens and nests with mixed inflammatory cell infiltration in a sclerotic stroma (original magnification × 200). (D) The tumors show mucus secretion in the lumens and eosinophils (original magnification × 200).

The patient underwent preoperative thyroid fine needle aspiration cytology examination. Cytology tests confirmed a diagnosis of thyroid papillary carcinoma. Intraoperative frozen section examination was also performed which confirmed the diagnosis of a malignant tumor. Thus, the patient underwent radical thyroidectomy for thyroid carcinoma. A gray-white and gray-red solid nodule with a diameter of 3.5 cm was found in the resected specimen (Fig. 1). The tumor was relatively well demarcated without a capsule. Under microscopic examination, the tumor exhibited 3 different morphological regions. There were multiple nodules which consisted of monocyte-like cells and scattered osteoclast giant cell-like multinucleated cells, resembling giant cell tumor of bone (Fig. 2A). Obvious nucleoli and mitotic figures were easily seen in the monocyte-like cells (Fig. 2B). Typical papillary thyroid carcinoma (PTC) was seen at the inner edge of the nodule and outside the nodule (Fig. 2A). Outside the above tumor area, another morphological area was found, which accounted for about 30% of the total lesion. The lesion in this area displayed an infiltrative pattern and composed of lumens and nests of cells lying in a sclerotic stroma infiltrated by eosinophils, small lymphocytes and plasma cells (Fig. 2C). The lumens contained colloid-like material or mucin (Fig. 2D). The nuclei exhibited mild to moderate nuclear pleomorphism and small nucleoli.

**Figure 3:**
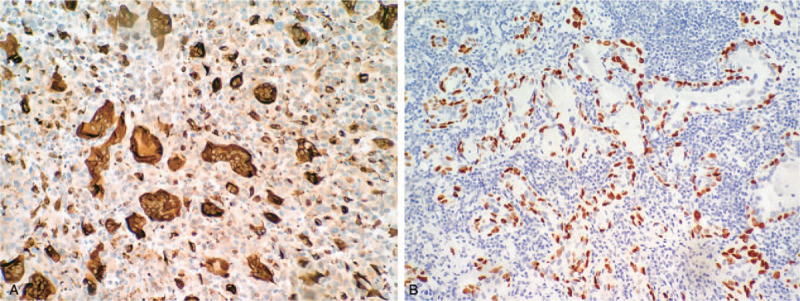
Immunohistochemical features of the tumor. (A) Osteoclast giant cell-like cells are positive for CD163, but monocyte-like cells are negative (original magnification × 200). (B) Sclerosing mucoepidermoid carcinoma with eosinophilia shows p63 positive staining for the outer layer cells (original magnification × 200).

**Figure 4:**
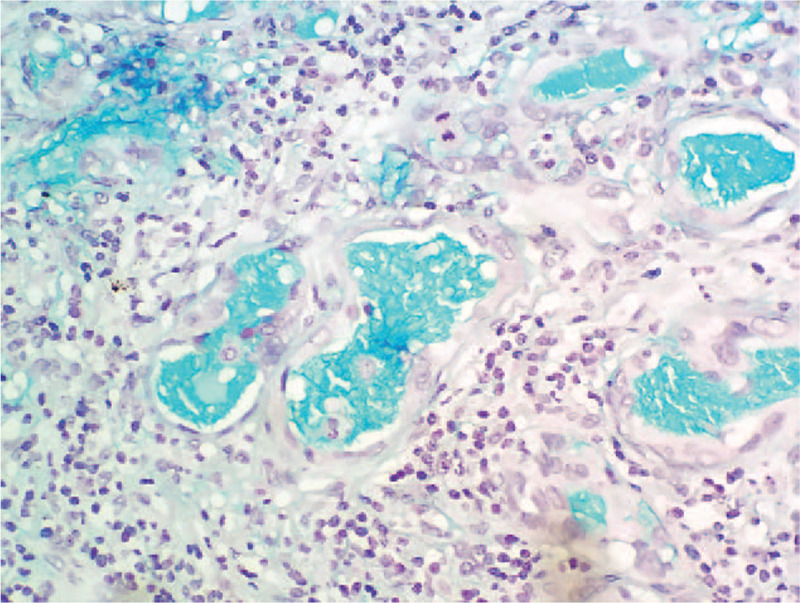
Sclerosing mucoepidermoid carcinoma with eosinophilia stained with Alcian Blue staining (original magnification × 400) shows positive staining for the mucus secretion in the lumens.

Immunohistochemical examination confirmed that monocyte-like cells were diffuse, with abundant expression of p63 and SATB2 but were negative for CD163, TTF-1, thyroglobulin (TG), AE1/AE3, cytokeratin (CK) 8, EMA and H3.3 G34W. The osteoclast giant cell-like multinucleated cells only expressed CD163 (Fig. 3A). The neoplastic cells of PTC expressed AE1/AE3, TTF-1, and PAX-8. The neoplastic cells of the third component were positive for PAX8 and negative for TTF-1. Immunostaining of CK7, CK8, p63, p40, and CK5/6 revealed a double-layer structure of the lumens and nests, that is, the inner layer cells expressed CK7 and CK8, the outer cells expressed p63 (Fig. 3B), p40 and CK5/6. Alcian Blue Staining showed positive reaction of the secretion in the lumens (Fig. 4). Using a micro-dissection approach, ARMS PCR confirmed that both PTC and the component resembling giant cell tumor of bone had BRAF^V600E^ mutation, but not the third component. A definitive diagnosis of ATC combined with sclerosing mucoepidermoid carcinoma with eosinophilia was made. There was no evidence of local recurrence or metastases during the 1 year and 3 months follow-up after the surgery. However, in this case, due to the high-grade histological changes, the prognosis was uncertain, and the patient was advised to undergo a further follow-up.

## 3 Discussion

Currently, ATC is often dedifferentiated from differentiated thyroid carcinoma, and the most common differentiated component is PTC.^[4]^ Our case had a component of typical PTC, which was mixed with the ATC component, suggesting that the ATC component was the dedifferentiated component of papillary carcinoma. ATC displays highly variable microscopic appearance, which can be mainly categorized into three patterns: sarcomatoid, giant cell, and epithelial cell differentiation.^[3]^ Osteoclast-like subtypes are very rare. In the current case, osteoclast-like giant cells were scattered in many monocyte-like cells. The monocyte-like cells had significant atypia and mitotic figures. These morphological features were consistent with the osteoclast-like subtype of ATC described previously.^[2,6]^ ATC usually lacks the expression of thyroid markers (TTF-1, TG) in immunophenotypes, while PAX-8 is expressed in 36% to 50% of ATC cases.^[2,3]^ Positive staining of CK indicates the epithelial nature of ATC, but negative staining for CK does not exclude the diagnosis of ATC. The main role for immunohistochemical examination of ATC is to rule out other undifferentiated malignant tumors, such as lymphoma and malignant melanoma.^[3]^ Since the monocyte-like cells in this case showed positive expression of p63 and SATB2 and negative expression of TTF1, TG, PAX-8, metastatic giant cell tumor of bone should be differentiated. An effective approach for differential diagnosis is radiological examination. In this case, no primary lesions of bone were found after systemic E-CT examination, providing evidence that the giant cell tumor-like component was primary thyroid tumor. Giant cell tumors of bone usually harbor histone H3.3G34W mutations, which can be detected by specific mutation antibodies.^[7]^ The specific H3.3G34W mutant antibody in this case was negative, and was different from that of giant cell tumor of bone. The frequent gene mutations in ATC include TP53 (54.4%), RAS (43%), BRAF (13.8%), PI3K-AKT pathway mutation (17%).^[4,8]^ The most common gene mutations in cases of ATC coexisting with thyroid papillary carcinoma are BRAFV600E (90%) and TERT promoter mutations (95%).^[9]^ In the current case, both ATC and PTC components had BRAFV600E mutation, further indicating that the ATC component was the dedifferentiated component of the papillary carcinoma.

Another component of this case was the thyroid sclerosing mucoepidermoid carcinoma with eosinophilia. Consistent with the literature, this case occurred in the background of chronic lymphocytic thyroiditis.^[10]^ Chan et al believed that the tumor may originate from follicular benign squamous cell nests metaplasia.^[5]^ The infiltrative nests and/or short strands of epithelial cells were seen under the microscope in the sclerosing stroma, with eosinophil, lymphocyte and plasma cell infiltration in the stroma. Few mucous cell differentiation and keratinization were seen in the epithelial cells. Immunohistochemical staining of p63 and TTF-1 showed variable results, and TG was persistently negative. Interestingly, some areas of this case showed a papillary growth pattern, a phenomenon never reported before. The main differential diagnosis of this case is Hashimoto thyroiditis with follicular epithelial squamous metaplasia. The squamous nests of Hashimoto thyroiditis generally lack significant eosinophil infiltration, epithelial nucleus enlargement, significant nucleolus, mitosis, mucus-secreting cells and mucus accumulation.^[11]^ No vascular and nerve infiltration was found in the tumor. Immunohistochemistry shows positive expression of TG in squamous nests.^[11]^ In our case, the cells of the nests showed nuclear atypia. Eosinophil could be easily identified. Mucus staining was positive while TG immunohisto-chemical staining was negative. These results indicated that it was a neoplasm, not a benign squamous metaplasia lesion. Thyroid sclerosing mucoepidermoid carcinoma with eosinophils is often negative for MAML2 suggesting different pathogenesis of salivary gland mucoepidermoid carcinoma.^[10,12]^ As a result, MAML2 FISH is not effective for differential diagnosis.

Osteoclast-like subtype of ATC and sclerosing mucoepidermoid carcinoma with eosinophil in the thyroid are extremely rare tumors, and even extremely rare for them to occur in 1 patient. This is the first case of this kind. During the 1 year and 3 months follow-up, no recurrence or metastasis was found in this case. However, given the aggressive nature of the ATC component, the prognosis of this condition may not be favorable. Sclerosing mucoepidermoid carcinoma with eosinophils, another component, is generally considered to be a low-grade malignancy. In recent years, however, it has been considered to be more aggressive.^[13]^ Therefore, the prognosis of this case is still uncertain and long-term follow-up is needed.

## Author contributions

**Data curation:** Lifang Shi, Wei Yan, Wenli Li, Baicheng Li.

**Supervision:** Xinjun Li.

**Writing – original draft:** Ruiqi Mao.
